# Understanding EFL Teacher Engagement in TDTs’ Collaborative Curriculum Design: A Chinese Case Study From the Activity Theory Perspective

**DOI:** 10.3389/fpsyg.2022.825274

**Published:** 2022-03-01

**Authors:** Zhonghua Wu, Jian Li, Le Cheng

**Affiliations:** ^1^School of Foreign Languages, Zhejiang University City College, Hangzhou, China; ^2^Department of Linguistics and Translation, School of International Studies, Zhejiang University, Hangzhou, China; ^3^School of Foreign Languages, Zhejiang Gongshang University, Hangzhou, China; ^4^Guanghua Law School, Zhejiang University, Hangzhou, China

**Keywords:** teacher engagement, collaborative curriculum design, activity theory, case study, EFL, TDT

## Abstract

While collaborative curriculum design (CCD) has gained increasing attention in the field of education, there is scant research as to how EFL teachers implement it in authentic design contexts. The present case study places particular emphasis on teacher activities during collaborative EFL curriculum development in China. The researchers adopt Activity Theory as an analytical tool to understand the relationships between EFL teachers, reform goals, and various aspects of the sociocultural context in which CCD is advocated, highlighting the pivotal role that mediation of knowledge/experience plays in the teacher learning community. Research findings indicate that the TDTs’ collaborative process is a socially constructed and culturally mediated activity that promotes EFL teacher development. Moreover, in-service teachers are prone to take challenges through educational reforms out of teacher motives and external stimuli. Limitations of the CCD model are also discussed to shed light on future research.

## Introduction

Since the reform and opening up in 1978, China has evolved into a country that owns the world’s largest population of English as a foreign language (EFL) learners in the new millennium (Wu, 2019). One of the reasons for this is that English is a compulsory course for students at all levels from elementary to college besides extensive public concern. However, recent years have witnessed some mismatches between the rapidly changing learning environment and the curriculum development, e.g., how to design an English course that satisfies student needs and social demands has aroused enormous attention among EFL teachers in Chinese tertiary institutions.

Teacher engagement is a multidimensional construct that reflects teachers’ participation and performance in fulfilling teaching-related roles through the allocation of physical, cognitive, and emotional energies (Perera et al., 2021). The main factors measuring teacher engagement in classrooms and schools in the Engaged Teachers Scale relate to cognitive, emotional, and social dimensions (including social engagement with students and colleagues, respectively; Klassen et al., 2013), which are generally used to illustrate how engaged teachers behave over time. As college English educational reform deepens across the country, especially against the release of the 2020 *Guidelines on College English Teaching*, Chinese EFL teachers feel obliged to optimize pedagogical practices through active engagement in reform. However, there has not been a consensus over the orientation of College English due to huge differences among universities and colleges. Some scholars (e.g., Wang, 2013; Hu and Xie, 2014) argue that College English should become instrumentally oriented and humanistically oriented, while others (e.g., Cai, 2017) suggest that it be used as a tool to facilitate EFL learners’ professional development. No matter which orientation a university favors, it is needed to integrate the goal into the talent cultivation process that is ensured by EFL courses.

Since curricula are more robust when designed through the collaborative engagement of teachers (Crites and Rye, 2020), the collaborative curriculum design (CCD) model tailored to local conditions is worth studying as a possible way to address the challenge mentioned above. CCD refers to a group activity of teacher designs teams (TDTs) that “usually focuses on student activities, lesson, modules, and courses” (Voogt et al., 2011, p. 1236). In other words, TDTs contribute collective professional expertise to designing the units of study to generate new ideas for instructional changes. Voogt et al. (2015) regarded the practice of CCD as effective to deepen teachers’ understanding of the subject matter and enhance the curriculum design competence. Bakah et al. (2019) confirmed that a collaborative professional development program serves the purpose of enhancing English teachers’ competence, e.g., communicative language teaching skills. But previous research findings also demonstrate that teachers with diverse professional backgrounds and specialized skills can either promote (Deketelaere and Kelchtermans, 1996) or hinder (Schneider and Pickett, 2006) curriculum development. Compared with the individually designed practice, CCD appears to be a highly complex and socially situated activity that is subject to multiple factors.

Based on Vygotsky’s model of mediation, the core of Engeström’s (2001) Activity Theory (AT) is the triangular structure of activity comprising six components, i.e., subject, object, instruments, rules, community, and division of labor. The theoretical framework is oftentimes adopted to analyze and understand human interactions based on the use of tools and artifacts. Although AT is not necessarily fit for all activities, it works well for some educational contexts (Bakhurst, 2009). As CCD is a process that involves the collaborative engagement of members in TDTs and is influenced by social interactions in educational settings (Jonassen and Rohrer-Murphy, 1999), the theory can be employed to demystify teacher activities during educational reforms and expound on the formation of instructional practices. Bleakley (2020) explored the curriculum development in medical education, arguing that the cultural-historical AT helps reconceptualize curriculum principles, e.g., integrating medical humanities into medical practices. Zahedi et al. (2017) investigated how a multidisciplinary team worked together to figure out a solution for designing a litter-disposal system, concluding that the conceptual framework could better outline the design process and account for the dynamics of collaboration. In reality, teachers promote professional development partly by cooperating with partners, which further triggers a sense of belonging to the shared community (Ronfeldt et al., 2015).

Given increased attention to CCD in financial literacy education (Compen and Schelfhout, 2021), biology teaching (Drits-Esser and Stark, 2015), and medical education (Bleakley, 2020), there is a paucity of research investigating EFL teachers’ engagement in collaboratively developing a course, though it is a widespread practice in Chinese elementary and secondary schools. According to Zhang (2021), EFL teachers in China’s higher education prefer working on their own in lesson preparation to working collaboratively with others. Therefore, it is necessary to probe into whether the CCD model is indeed inferior to the individual lesson preparation. To fill in the gap, the present study endeavors to answer the following research questions with a particular focus on the process of Chinese EFL teachers’ collaborative curriculum design at a local university: (1) Do EFL teachers benefit from their engagement in CCD?; (2) How do EFL teachers engage themselves in CCD?; and (3) To what extent is EFL teacher engagement in CCD reflected in pedagogical practices? Based on related discussions, this study will contribute to the existing literature on teacher learning and development.

## The Present Study

### The CCD Project and the Participants

As the university underwent a transition from an independent college to a public university, it has adjusted the talent training agenda, targeting the cultivation of students’ application abilities and global vision. The Production-Oriented Approach (POA), which is aimed at removing the segregation between learning and application by means of motivating, enabling, and assessing (Wen, 2015), was implemented to keep consistent with the school orientation and EFL teachers’ expectations. More broadly, the research is conducted under national guidelines that call for EFL teachers to update teaching approaches and means of instruction (e.g., blended learning) to promote learning outcomes. Considerations of the above contextual factors, both local and national, have underpinned the adoption of the CCD model initiated in 2020, which was actually framed around a larger research project on college English educational reform in China.

When the head of the College English Teaching Department shared her reform proposal at a routine meeting, 16 course instructors volunteered to sign up for it. The participants were teaching first-year non-English majors at the time of the study, equipped with rich pedagogical knowledge and strong professional self-identity. They were divided into four groups in charge of task design, courseware preparation, technical support, and language assessment, respectively. Their demographic information is illustrated in Table 1. As higher scores in National College Entrance Exam are deemed as an indicator of better academic achievements, the newly enrolled undergraduate students were believed to be competent for tasks in the POA mode.

**Table 1:** EFL teachers’ demographic information.

Number of teachers	Gender	Years of teaching	Course name	Target students	For or against POA
11	Female: 10 Male:1	<10–≤15	College English Band 3	First-year Non-English Majors	F
2	Female: 2 Male: 0	<15–≤20	College English Band 3	First-year Non-English Majors	F
1	Female: 0 Male: 1	<20–≤25	College English Band 3	First-year Non-English Majors	F
2	Female: 2 Male: 0	<25–≤30	College English Band 3	First-year Non-English Majors	F
Total: 16					

### Data Collection and Analysis

The data for the case study were collected from two sources, i.e., participant observation and semi-structured interviews. Participant observation is frequently used as a crucial instrument for data collection in diverse disciplines (Kawulich, 2005), especially social psychology and cultural anthropology, because it enables researchers to observe, engage in, and reflect upon participants’ activities in a real-life setting. The first author worked as a key member of TDTs and witnessed how the CCD model evolved throughout. He took responsibility for language assessment, strictly followed the POA approach in his teaching, and attended project meetings on a regular basis. However, problems related to participant observation may include issues such as heavy reliance on critical informants, individual preference for specific themes, and how to balance the dual roles between an observer and a participant. As Kawulich (2005) notes, research validity becomes stronger if other qualitative research methods are integrated into the observation process, such as interviews and surveys. To address subjectivity arising from the above, the other two authors helped design an interview protocol based on the first author’s field observation notes and self-reflection. Various teacher groups were later interviewed to triangulate the account.

**Figure 1:**
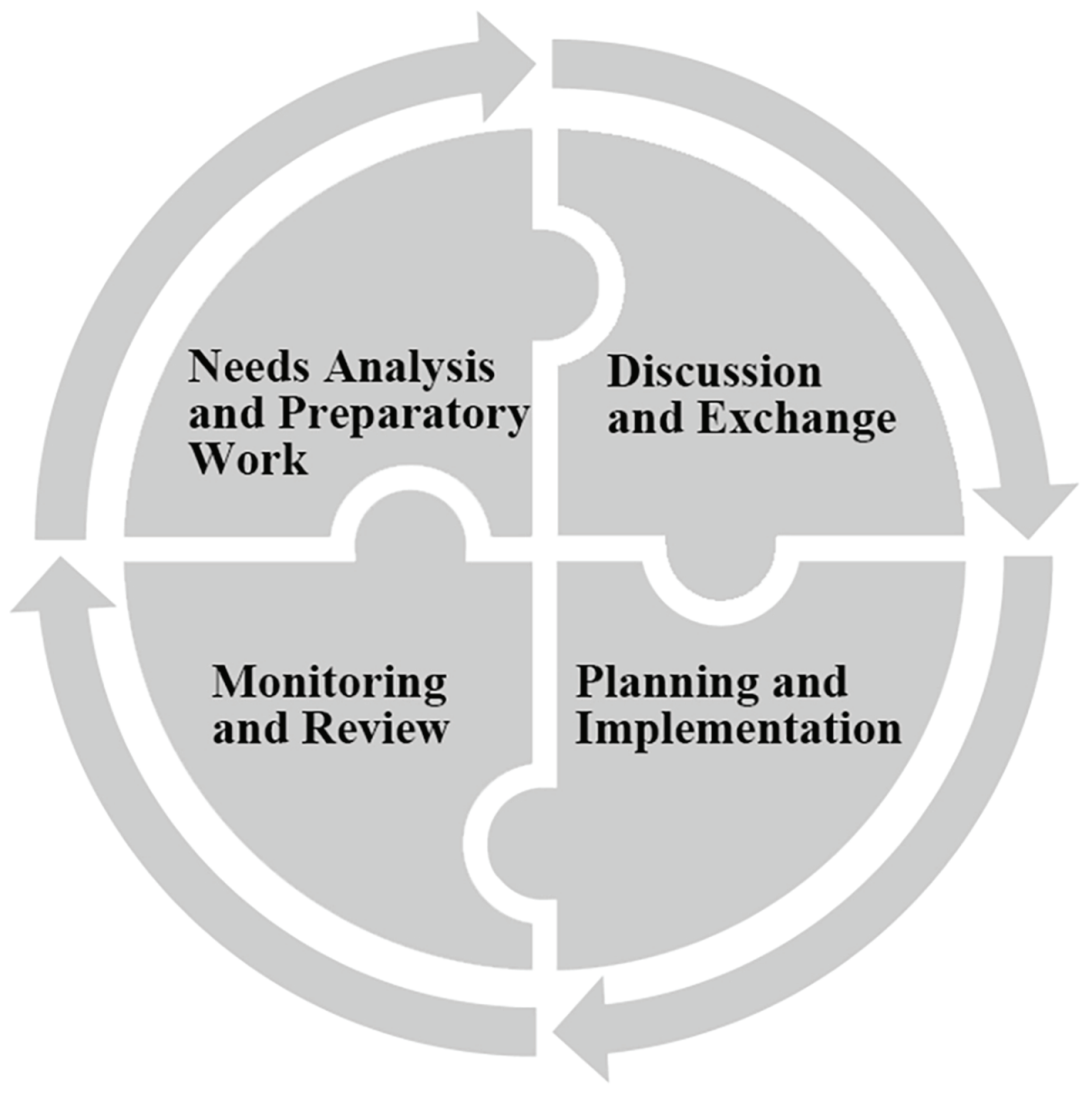
Four critical stages in the CCD Model.

As indicated in Figure 1, the iterative design process consists of four stages: student/teacher needs analysis, discussion and exchange of ideas, planning and implementation of CCD, and monitoring and review of overall work. Nevertheless, the CCD model (collective) and the traditional lesson preparation (individual) were implemented alternately in the two semesters due to teaching requirements by the department, which offers an excellent opportunity to make comparisons of distinct design modes. Given that all participants committed to the coordinated development of the course, we attempted to keep track of EFL teacher activities manifested in their professional practices across the board.

Using a systematic qualitative inductive analysis (Strauss and Corbin, 1998), we examined the data obtained from observations during the four critical stages and online/offline semi-structured interviews in the academic year. We also shared the preliminary findings with some EFL teachers and made revisions for data interpretation in accordance with relevant feedbacks.

## Results

The major findings of the study under three central themes are presented as follows: (1) EFL teachers’ perception of their engagement in CCD as a reciprocal process, (2) EFL teachers’ cognitive, social, and emotional engagement in CCD, and (3) discrepancies existing between teachers’ expectations and the reality. In this section, they are reported with data from interviews (Int) and field observation notes (FON).

### EFL Teachers’ Perception of their Engagement in CCD as a Reciprocal Process

Although EFL teachers were assigned different roles, such as task design, courseware preparation, technical support, and language assessment, they mostly worked together across the four critical stages so that suggestions and comments from each sub-community could contribute to fulfilling the holistic reform target. The CCD turned out to be beneficial for each participant in the four groups. Through mutual communication and effective discussion, the shared “intellectual products,” such as course wares and quizzes, have greatly boosted EFL teachers’ working efficiency and sharpened their teaching skills.

T1, a female teacher from the task design group, taught College English in two different modes across the 2020–2021 semester. From the first excerpt, we know that she was quite satisfied with her engagement in CCD because she profited from collective wisdom contributed by her colleagues, e.g., saving the trouble of making course wares by herself. Unlike the individual lesson preparation, CCD seems more efficient and manageable in that the workload is broken down with each individual doing his share of duty. Specifically, the design group outlined how a learning module should be implemented and prepared supplementary materials for reference, while EFL teachers in other groups could use them effectively and confidently in classroom practices by navigating through the well-organized syllabus; for instance, they did not need to rack the brains about class activities.

### Excerpt 1

By designing three units of study in the syllabus, I’m able to dig deep into the knowledge points and gain a better understanding of teaching content, which further promotes my competence in classroom teaching. This also means I save the trouble of preparing course wares that are in the charge of other teachers. When browsing through the PPTs, I find them very informative and inspiring. To my satisfaction, my teaching effectiveness is much better than before. (T1, Int)

Moreover, T2 points out that frequent interactions with more experienced teachers facilitate her teaching process and build intimacy among EFL teachers. It is also worth noting that L1, EFL, textbooks, and websites play an important supporting role for EFL teachers to realize reform goals.

### Excerpt 2

Honestly speaking, I didn’t know what I could obtain from my engagement into CCD at first. But I’m sure I should participate in the project actively because I want to make a change to my way of teaching, which seems to be old-fashioned. I experiment with different teaching principles, communicate with competent colleagues in L1 and EFL, and improve my teaching outcomes by delving into the textbook and reference materials. The collaboration lets us make progress jointly and deepen mutual understanding, especially when we cope with tricky issues. (T2, Int)

T1 strictly followed the POA in the first semester and observed better language output of her students. Although EFL learners were constantly busy with output-oriented tasks including English debate, film-dubbing, drama play, and essay writing, they did make varying progress, such as developing language abilities and promoting cultural awareness. This formed a stark contrast to her instruction in the second semester when the traditional way of teaching was implemented. In brief, the CCD model is not only good for any participant in and out of TDTs, but for EFL learners who benefit from the teaching reform.

### EFL Teachers’ Cognitive, Social, and Emotional Engagement in CCD

Before the reform, EFL teachers conducted a needs analysis to balance the interests of all parties (e.g., students, teachers, and university authorities) by means of teaching reflection, literature research, and interviews. The subsequent teacher activities are guided by motives that greatly affect CCD, as argued by Yu and Lee (2015). According to T3, EFL teachers are confronted with huge pressure in the context of reduced credits for College English courses nationwide; as a result, they feel highly motivated to undertake the reform to guarantee the quality of talent cultivation and professional values as well. From an analytical perspective, teacher activities are found to be driven by motives that are shaped and mediated by the sociocultural context.

### Excerpt 3

Nowadays, college English teachers are placed under great pressure, and we need to choose whether to reform or perish. The fact that credits for English courses have been reduced nationwide makes me feel quite stressed. As the school enters into a new stage of development, most of us don’t want to lag behind. Personally, I take great interest in educational reform, which, I believe, is good for my career as an EFL teacher. (T3, Int)

The internal and external stimuli motivate EFL teachers to get out of the comfort zone and engage in goal-oriented activities in the teacher learning community. During the reform, they are constrained by pedagogical theories to identify with, national guidelines on EFL teaching, and teaching norms of the school; yet, how to fully integrate them into educational reforms is of vital significance. The data analysis shows that many technical problems concerning design principles were solved through professional guidance of the POA expert team, demonstrating that mediation of knowledge/experience among teachers, experts, and even administrative staff is one of the driving forces behind reform, as the following filed note reveals.

### Excerpt 4

At a regular meeting, the department head motivated all participants to change mindsets to fit into the educational reform. She informed us that the design group has clarified how to effectively use the teaching approach for instructional practices by consulting experts and called for everyone present to seek help from them when practical problems occur. Besides, the work progress of TDTs was shared and discussed in the meanwhile. (FON)

The CCD model also involves regular reflection on teacher activities, which is conducive to each individual’s performance. There is no doubt that peer feedbacks may lead to further refinements of tasks. But EFL teachers have to deal with criticism from their partners in TDTs if there is still room for job improvement. After negotiating positively with one another, they tend to become emotionally stronger and mentally sharpener. As T4 reports, “I felt stressed out when my work was challenged by others; but the collaboration toward shared goals forced me to build a stronger mind and become more critical, which is a good thing.” (T4, Int).

Overall, the reform primarily relates to EFL teachers’ cognitive, social, and emotional engagement in CCD under the influences of teacher motives, mediation of knowledge, and strong emotion, which are considered as key factors affecting teacher activities in curriculum development.

### Discrepancies Existing Between Teachers’ Expectations and the Reality

In the first two stages, i.e., needs analysis and group discussion, all participants familiarized themselves with the educational ideas and group responsibilities and moved toward the goal as scheduled. But some different voices in TDTs began to emerge when it came to the implementation stage.

T5, who was a distinguished EFL teacher, kept a reserved attitude toward the ongoing curriculum reform because she noticed some discrepancies between her expectations and the reality, such as the inappropriate choice of sentence examples for word study, heavy reliance on the teacher’s manual for sentence learning, and poor layout design in PowerPoints. This mainly results from the inertia and irresponsibility of some teachers who rushed to get the courseware preparation done while neglecting potential influences of their performance on overall curriculum development. When it comes to group activities, individual shared responsibility needs to be fulfilled as required; otherwise, the collective work could not live up to expectations.

### Excerpt 5

I think collaborative design is a good practice because it’s time-saving and more goal-oriented. But when I used the course wares in my class, I found some parts in them were not satisfying. For example, regarding word study, a few sentence examples were copied directly from the dictionary without thorough consideration of their appropriateness for students, and the analysis of long and complicated sentences proved to be superficial, so I made necessary adjustments. I’m also in favor of combined teaching approaches and strategies that are suitable for my class. (T5, Int)

As T6 reports, the low level of English proficiency created a hindrance for her students to take an active role in challenging activities. Therefore, she adjusted class arrangements to help learners remain enthusiastic about English. The above findings suggest that context-specific factors need to be appropriately addressed.

### Excerpt 6

The language proficiency of my students is not as good as expected. Some tasks in the syllabus (e.g., English debate) are quite challenging for them. Thus, I didn’t follow what had been stipulated by the design group and redesigned some class activities. The reason for this is that I don’t hope my students feel frustrated and lose interest in learning English. Despite the standardized class management, I sometimes prefer to keep my own teaching styles. (T6, Int)

Furthermore, due to limited class hours, EFL teachers are found to pay substantial attention to the development of students’ application ability while simplifying procedures for imparting knowledge in lectures, as evidenced by the following excerpt. After realizing such a problem, the department head decided to reduce the number of learning modules from six to five in the new semester.

### Excerpt 7

It was noticed that some teachers spent a large amount of time preparing students for various tasks at the expense of a detailed explanation of knowledge points. In the second half of the semester, they struggled to catch up with the teaching progress. Therefore, how to allocate learning hours for each module should be brought to the forefront. At the end of the semester, our department collected teacher feedbacks on the reform and discussed suggestions for improvement. (FON)

### Discussion and Implications

**Figure 2:**
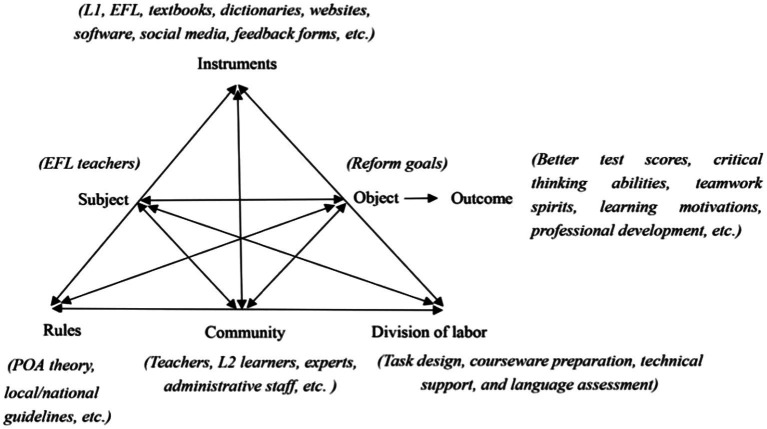
Activity theory as an interpretive framework for teacher activities in CCD.

Zahedi et al. (2017) argue that collaborative design is intertwined with collaborative learning. Nevertheless, previous research attaches more importance to the collaborative learning of students than that of EFL teachers, indicating the latter is a less-explored area of study. Figure 2 illustrates how teacher activities in the CCD model are constructed by drawing on the AT framework. As is clearly depicted, the process involves three critical elements: a subject, an object, and instruments. The subject (EFL teachers) and the object (reform goals) are linked through mediating artifacts, such as L1, EFL, textbooks, dictionaries, websites, software, social media, and feedback forms. The relationship between the subject and the community is mediated by rules that EFL teachers abide by, e.g., pedagogical theories, teaching guidelines, and school requirements, while the relationship between the object and the community is mediated by a division of labor that specifies how tasks are allocated among participants, i.e., different roles and responsibilities in TDTs. It is safe to say that AT provides an analytical lens for us to understand the relationships between EFL teachers, reform goals, and various aspects of the sociocultural context in which CCD is advocated.

The above diagram represents a typical model of EFL teachers’ collaborative learning. Since design activities could promote teacher professional development through collaboration and consultation, this study echoes Zhang et al.’s (2018) research finding that collaborative lesson preparation boosts the quality of instructional practices, as evidenced by enhanced teaching competence and student language output. Other advantages for CCD may include standardizing teaching management, improving working efficiency, and deepening mutual understanding, but most of all, the thoroughly designed model is more systematic, scientific, and efficient than the individual lesson preparation. The Chinese cultural tradition of collectivism underlies the practice, bringing teaching experience and teacher effectiveness into full play. However, building a successful team is more than merely asking teachers to work together, but tapping into the expertise of every individual to achieve a greater good.

The findings of this study show that the CCD model relates to the participants’ social, cognitive, and emotional engagement in addition to physical engagement. On the social dimension, EFL teachers acquire new knowledge and skills from colleagues with respect to task arrangements, selection of course materials, teaching strategies, and so on. Vygotsky’s (1978) zone of proximal development (ZPD) can elucidate the social process of knowledge mediation, i.e., bridging the gap between what an EFL teacher cannot achieve unsupported (current development level) and what he or she can do with guidance or in collaboration with competent peers (potential development level). Although EFL teachers’ professional knowledge and teaching skills vary from person to person, the CCD model offers them opportunities for reflective thinking and social dialogue (Oxford, 1997), e.g., negotiating about the usages of modality within diverse sociocultural contexts (Cheng and Cheng, 2014). When the units of study are designed collaboratively, the cohorts of teachers could profit from a diversity of knowledge, beliefs, values, and experiences, making them aware of the potential of individual professional development through curriculum innovations.

From the perspective of cognitive psychology, EFL teachers are motivated by a strong belief that engaging in educational reforms is indispensable for professional advancement. For example, one EFL teacher, whose primary research interest is EFL teaching with a particular focus on L2 speaking, volunteered to take charge of the oral English assessment, showing that teaching and researching can be fully integrated to achieve mutual benefits. In accord with Carson and Chase’s (2009) finding, teacher motives are regarded as a key factor affecting the smooth implementation of curriculum development. Besides, teachers from the same department are liable to treat teacher-to-teacher interactions as a way to strengthen bonds of unity so as to better cope with teaching challenges from various facets of society. While previous research (Schneider and Pickett, 2006) has found that specialized language and disciplinary differences among partners in TDTs could create hindrances for curriculum development, the present study suggests that shared professional backgrounds contribute to the synergetic development of participants in college English curriculum development.

Despite the prominent advantages of CCD, not all EFL teachers feel satisfied with it. One teacher informed us that she did not base her lesson entirely on the recommended teaching approach because she preferred mixed ones. This does not deny the significance of POA in improving students’ language application ability but implies that teachers as an organizer and a facilitator of EFL learning can opt for the way knowledge is imparted to accomplish curriculum objectives. Course instructors should be allowed to tailor EFL teaching to local conditions at their discretion, for one of the aims of educational reforms is to probe into the mode of teaching, which should be contextually dependent, negotiable, and dynamic. Likewise, it is critical to examine what countermeasures should be undertaken to compensate for deficiencies in current teaching practices. For instance, EFL teachers could raise awareness about educational reforms through regularly scheduled seminars, where demo classes can be presented, discussed, and reflected. This seems advantageous for teachers who are reluctant to experiment with new approaches due to fossilized teaching styles, limited energy, and unfamiliarity with pedagogical theories. On top of this, unremitting efforts should be made to address teacher/student needs and other practical concerns appropriately.

Below are two practical implications drawn from the aforementioned findings. For one thing, the CCD model entails highly motivated teachers who adapt themselves to the ever-changing teaching environment as well as a harmonious working atmosphere where teachers are offered professional development opportunities through knowledge mediation. This can be made more desirable through incentive policies and team building. For another, teacher professional development is an ongoing process that should not come to a halt at the end of educational reforms. EFL teachers are supposed to frequently strengthen contacts with competent peers and specialists in and out of the university and improve the design model based on contextual conditions.

## Conclusion

The TDTs’ collaborative process is a socially constructed and culturally mediated activity that promotes EFL teacher learning and development through shared knowledge. The CCD model under discussion has numerous advantages, such as standardizing teaching management, improving working efficiency, and deepening mutual understanding, but it may, to some extent, run the risk of losing EFL teachers’ individual characteristics. More importantly, dynamic adjustments to local conditions should be regularly made to suit instructional practices, suggesting that context-specific factors need to be cautiously addressed.

This exploratory study also indicates that in-service teachers are prone to take challenges through educational reforms out of teacher motives as well as external stimuli. Teacher learning provides a great impetus for educational innovations. When teachers actively engage in activities that lead to a change in knowledge, beliefs (cognition), and instructional practices (behavior; Bakkenes et al., 2010), their educational innovations can be further promoted. While our research findings cannot be utterly generalizable to CCD models in other pedagogical situations, they still contribute to a better understanding of the relationships between EFL teachers, reform goals, and various aspects of the sociocultural context in which CCD is advocated, especially the pivotal role that mediation of knowledge/experience plays in a Chinese EFL teacher learning community.

## Data Availability Statement

The raw data supporting the conclusions of this article will be made available by the authors, without undue reservation.

## Ethics Statement

The studies involving human participants were reviewed and approved by Zhejiang University City College. The ethics committee waived the requirement of written informed consent for participation.

## Author Contributions

ZW designed the research and wrote the initial draft of this article. JL assisted in the data analysis and proofread the final version. LC made many constructive comments on the earlier versions of this article. All authors contributed to the article and approved the submitted version.

## Funding

This work was supported by the Educational Reform Project of Zhejiang University City College (grant number: JG1812).

## Conflict of Interest

The authors declare that the research was conducted in the absence of any commercial or financial relationships that could be construed as a potential conflict of interest.

## Publisher’s Note

All claims expressed in this article are solely those of the authors and do not necessarily represent those of their affiliated organizations, or those of the publisher, the editors and the reviewers. Any product that may be evaluated in this article, or claim that may be made by its manufacturer, is not guaranteed or endorsed by the publisher.
